# PMIndoor: Pose Rectified Network and Multiple Loss Functions for Self-Supervised Monocular Indoor Depth Estimation

**DOI:** 10.3390/s23218821

**Published:** 2023-10-30

**Authors:** Siyu Chen, Ying Zhu, Hong Liu

**Affiliations:** 1 Institute of Artificial Intelligence, University of Science and Technology Beijing, Beijing 100083, China; csyunling@stu.pku.edu.cn; 2 Key Laboratory of Machine Perception, Shenzhen Graduate School, Peking University, Shenzhen 518055, China; hongliu@pku.edu.cn

**Keywords:** deep learning, indoor monocular depth estimation, self-supervised learning, multiple loss functions, pose rectified network

## Abstract

Self-supervised monocular depth estimation, which has attained remarkable progress for outdoor scenes in recent years, often faces greater challenges for indoor scenes. These challenges comprise: (i) non-textured regions: indoor scenes often contain large areas of non-textured regions, such as ceilings, walls, floors, etc., which render the widely adopted photometric loss as ambiguous for self-supervised learning; (ii) camera pose: the sensor is mounted on a moving vehicle in outdoor scenes, whereas it is handheld and moves freely in indoor scenes, which results in complex motions that pose challenges for indoor depth estimation. In this paper, we propose a novel self-supervised indoor depth estimation framework-**PMIndoor** that addresses these two challenges. We use multiple loss functions to constrain the depth estimation for non-textured regions. We introduce a pose rectified network that only estimates the rotation transformation between two adjacent frames of images for the camera pose problem, and improves the pose estimation results with the pose rectified network loss. We also incorporate a multi-head self-attention module in the depth estimation network to enhance the model’s accuracy. Extensive experiments are conducted on the benchmark indoor dataset NYU Depth V2, demonstrating that our method achieves excellent performance and is better than previous state-of-the-art methods.

## 1. Introduction

Through the visual system, humans acquire information about the external world and can perceive and judge the surrounding environment accurately. Computer vision technology, which aims to enable computers to have the ability to perceive the external environment like humans, has become a significant topic in the current field of computer research. Depth estimation is a very important problem in the field of computer vision, and it has a wide range of applications, such as intelligent robots [1], 3D reconstruction [2,3], autonomous driving [4], augmented reality [5], etc. Deep learning technology has brought great advantages to depth estimation. It not only has lower requirements for hardware devices and environmental conditions, but is also convenient and flexible to implement with high accuracy. Eigen et al. [6] introduced a novel approach to monocular depth estimation by utilizing a supervised learning methodology. Their method employed a convolutional neural network architecture that integrated both global and local depth information. This constituted the inaugural implementation of deep learning methodologies in addressing the challenges of monocular depth estimation. Numerous supervised methods [7,8,9,10,11,12] have been proposed for monocular depth estimation subsequently. To make effective use of large amounts of relatively cheap label-free data to improve learning performance, self-supervised methods have emerged. Garg et al. [13] proposed a self-supervised convolutional network trained using the color consistency loss between stereo image pairs as a supervised signal. Godard et al. [14] proposed a left–right depth consistency loss to train self-supervised networks. However, most of the existing depth estimation methods [15,16,17] are designed for outdoor scenes such as cities, campuses, and roads, and have limited applicability to indoor scenes, which have been relatively less explored and have unsatisfactory results compared to outdoor situations. This is due to the fact that indoor scenes are complex, dense, highly continuous, and computationally demanding, as well as lack distinctive global or local features. Typically, the challenges and difficulties of indoor monocular self-supervised depth estimation can be summarized as follows: (1) Structure priors: objects in indoor scenes have less structural regularity compared to those in outdoor scenes, such as the sky, roads, etc. In indoor scenes, objects are arranged in a disorderly manner, which poses a great challenge for depth estimation. (2) Challenging lighting conditions: indoor scenes have more complex lighting conditions than outdoor scenes, such as dark areas, reflective surfaces, etc. These complex lighting conditions make it difficult to obtain accurate depth information. (3) Non-textured regions: indoor scenes often contain some non-textured or low-textured regions, such as walls, ceilings, etc. These regions can affect the commonly-used photometric loss function for self-supervised monocular depth estimation and can lead to erroneous estimation. (4) Camera pose: in outdoor scenes, sensors are usually fixed on moving vehicles, and pose estimation usually only involves three degrees of freedom; however, in indoor scenes, handheld cameras are often used and sensors can move arbitrarily, resulting in more complex motions, which undoubtedly brings challenges to indoor depth estimation.

In recent years, some indoor depth estimation methods have also emerged. Zhou et al. [18] proposed a new optical-flow-based training paradigm which handles the non-textured regions. Yu et al. [19] proposed a novel technique that leverages distinctive keypoints, patch-level warping, and superpixel-based regularization to cope with non-textured regions. Li et al. [20] leveraged structural regularities and integrated normal estimation and planar region detection as auxiliary tasks to deal with these problems. Ji et al. [21] proposed two novel modules for depth and pose estimation: a depth factorization module that handles the rapid scale changes in the depth network, and a residual pose estimation module that mitigates the inaccurate rotation prediction in the pose network, resulting in improved depth prediction. Bian et al. [22] argued that the rotation between consecutive frames is a source of noise that affects the training process. Therefore, they suggested a rectification step to eliminate the rotation. We share the same observation with Bian et al. [22] and adopt the same strategy. However, we improve upon their work by further modifying the network architecture and taking into account the effect of non-textured regions in indoor scenes. The experimental results show significant improvements. In the following, we will elaborate on our work.

**Figure 1:**
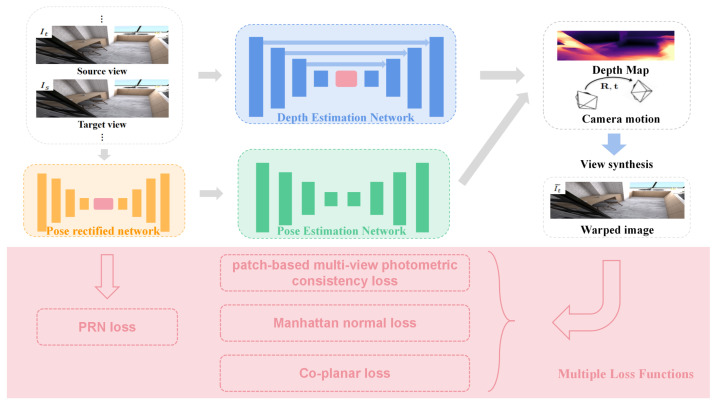
Overview of the proposed **PMIndoor**. **Depth estimation network**: we use a U-Net framework, an encoder–decoder network with skip connections, and insert multi-head self-attention modules (MHSA) to improve the accuracy of the model. **Pose estimation network**: we employ an encoder–decoder structure to estimate the camera motion between two frames. **Pose rectified network (PRN)**: we introduce a pose rectified network (PRN) before the pose estimation network to remove the rotational motion between adjacent frames. **Multiple loss functions**: we use multiple loss functions including patch-based multi-view photometric consistency loss, Manhattan normal loss, Co-planar loss, PRN loss, etc., to solve the camera pose problem and the non-textured regions problem.

In this paper, we propose **PMIndoor**, a self-supervised monocular depth estimation framework, as shown in Figure 1. Our proposed model framework is mainly designed to address two issues in indoor depth estimation: (i) non-textured regions, and (ii) camera pose. Regarding the non-textured region problem, indoor scenes usually have many non-textured regions, such as ceilings, walls, floors, etc. These regions often cause problems for the commonly-used point-based photometric loss, because these regions usually have similar values that lead to erroneous point matching. Therefore, we use multiple loss functions to solve this problem. First, we employ the patch-based multi-view photometric consistency loss proposed in P^2^net [19], which uses local patches instead of point-based methods to obtain photometric loss, thus having better discriminability and accuracy for indoor scenes. Second, we introduce two loss functions proposed in Structdepth [20]: Manhattan normal loss and Co-planar loss, which use the structural regularity information of indoor scenes to attain additional supervision information to solve the problem of non-textured regions in indoor scenes. The main idea of Manhattan normal loss is to align the normal vectors predicted from the depth map estimated from the main planes (walls, ceilings, floors, etc.) with the dominant directions extracted from the image vanishing points, and the discrepancy constitutes the Manhattan normal loss. Co-planar loss is to first perform plane region detection, and then unify the points that are located on the same plane to the same plane, and compute the loss as Co-planar loss. Regarding the camera pose problem, indoor scenes (usually captured with handheld devices) have more rotational motion compared to outdoor scenes (where sensors are usually fixed on vehicles), resulting in pose estimation that is more difficult and inaccurate. In the paper SC_Depthv2 [22], the authors demonstrate through rigorous mathematical derivation that rotational motion is irrelevant to depth estimation. Namely, if the rotational motion cannot be accurately estimated, it will introduce a lot of noise to depth estimation. Therefore, we propose the Pose Rectified Network (PRN), which is used to eliminate the rotational motion between adjacent frames, to improve the accuracy of the model. And we introduce an additional supervision signal, PRN loss, to constrain the training and to remove the rotational motion between adjacent frames as much as possible. Furthermore, we incorporate multi-head self-attention modules (MHSA) into the depth estimation network to improve the accuracy of the depth estimation. Multi-head self-attention modules can overcome the limitation of the local receptive field of convolutional neural networks, achieve global perception, and improve the capacity for modeling of long-distance dependence and global correlation in images. At the same time, they can make the model pay attention to multiple key regions simultaneously, let the model extract different semantic information in different representation subspaces, improve the feature capture ability of different positions and scales in images, and enhance the model’s expression and generalization ability. We conduct extensive experiments on the indoor benchmark dataset NYUv2 [23], and the experimental results show that our method **PMIndoor** outperforms many previous state-of-the-art methods.

Our contributions can be summarized as follows:

- We propose a new pose rectified network (PRN) to solve the camera pose problem, while also using the pose rectified network loss to remove the rotational motion between adjacent frames.
- We use multiple loss functions, such as patch-based multi-view photometric consistency loss, Manhattan normal loss, and Co-planar loss, to solve the problem of non-textured regions.
- We add multi-head self-attention (MHSA) modules to the depth estimation network to improve the expression and generalization of the model.
- The experimental results on the indoor benchmark dataset NYUv2 [23] demonstrate that our method **PMIndoor** outperforms many existing state-of-the-art methods.

## 2. Method

In this section, we introduce the self-supervised monocular depth estimation framework **PMIndoor** proposed in this paper. We first provide an overview of our framework. Then, we explain three core components: depth estimation network, pose rectified network, and multiple loss functions, in detail.

### 2.1. Overview

The self-supervised monocular depth estimation framework for indoor scenes designed in this paper is shown in Figure 1. Our framework consists of four components: depth estimation network, pose estimation network, pose rectified network and multiple loss functions. We use a five-frame (one target frame, 4 source frames) input, which is fed into the depth estimation network and the pose estimation network, respectively. The depth estimation network adopts the U-Net architecture, an encoder–decoder network with skip connections, to estimate the dense depth map. The pose estimation network employs an encoder–decoder structure to estimate the camera motion between two frames. Moreover, we introduce a pose rectified network (PRN) before the pose estimation network to address the camera pose problem. We also incorporate a multi-head self-attention (MHSA) module into the depth estimation network to improve the model’s accuracy. For the loss functions, we use multiple loss functions including the patch-based multi-view photometric consistency loss, Manhattan normal loss, Co-planar loss and PRN loss, etc., to enhance the model’s performance and tackle the challenge of non-textured regions and the camera pose problem.

### 2.2. Depth Estimation Network

**Figure 2:**
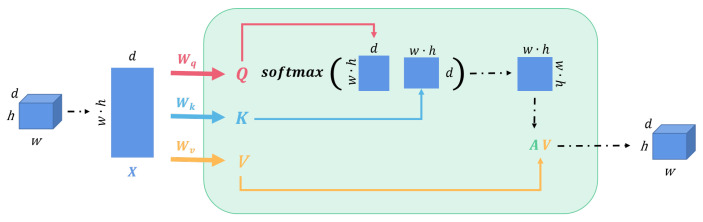
Structure of the multi-head self-attention (MHSA). The input tensor is transformed into the corresponding query (*Q*), key (*K*), and value (*V*), and then fed into the MHSA for learning. *A* is computed from *Q* and *K*.

**Figure 3:**
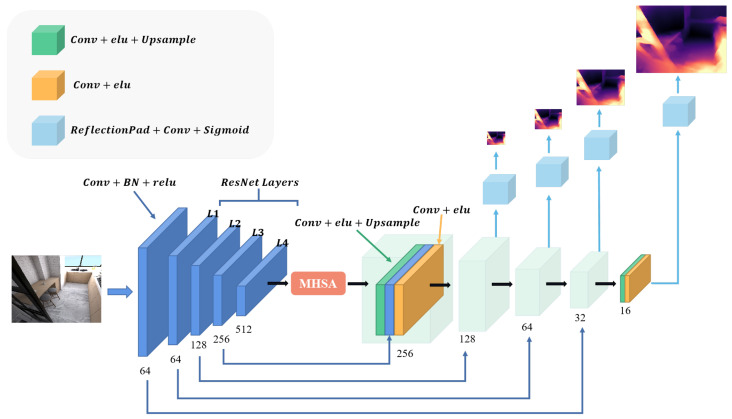
Structure of Depth Estimation Network. The input is an RGB image, and the output is four depth maps of different scales. The network is an encoder–decoder architecture with skip connections, and a multi-head self-attention module (MHSA) is inserted in the middle to improve the accuracy of the depth estimation.

The depth estimation network used in this paper is based on the U-Net architecture, a typical encoder–decoder network. The basic structure follows Monodepth2 [17], and skip connections are added in between to estimate the dense depth map. Moreover, we insert a multi-head self-attention module (MHSA) between the encoder and the decoder. Multi-head self-attention modules allow the model to focus on multiple key areas simultaneously, enabling the model to obtain different semantic information in different representation subspaces, enhancing the attainment of features at different positions and scales in the image, and optimizing the model’s expressive and generalization abilities. At the same time, it can break the limitation of the local receptive field of convolutional neural networks, achieve global perception, and improve the modeling ability of long-distance dependence and global correlation in the image. The specific network structure is illustrated in Figure 2. We employ a four-head self-attention module. The high-dimensional features extracted by the encoder are projected as the query (*Q*), key (*K*), and value (*V*), and are fed into the MHSA module for training, as illustrated in Figure 3. This process can be formally described as follows,

(1) $$Attention\left(Q,K,V\right)=softmax\left(\frac{QK^{T}}{\sqrt[{}]{d_{k}}}\right)V=AV.$$

We also follow the same practice as Monodepth2 [17] regarding the output of the depth estimation network, which produces four different scale depth maps to construct the photometric loss, as illustrated in Figure 2.

### 2.3. Pose Rectified Network

This paper introduces the pose rectified network (PRN), which aims to eliminate the rotational motion between consecutive frames and improve the model accuracy for the camera pose problem. The SC_Depthv2 [22] mathematically proves that the rotational motion and the depth estimation results are independent. Therefore, an inaccurate estimation of the rotational motion will introduce significant noise to the depth estimation. Based on this theory, we propose a novel PRN network that is integrated into the existing depth estimation framework to estimate the rotational motion between consecutive frames. We then apply a transformation projection using the estimated rotation to eliminate the rotational motion between the frames, which may otherwise cause more errors.

**Figure 4:**
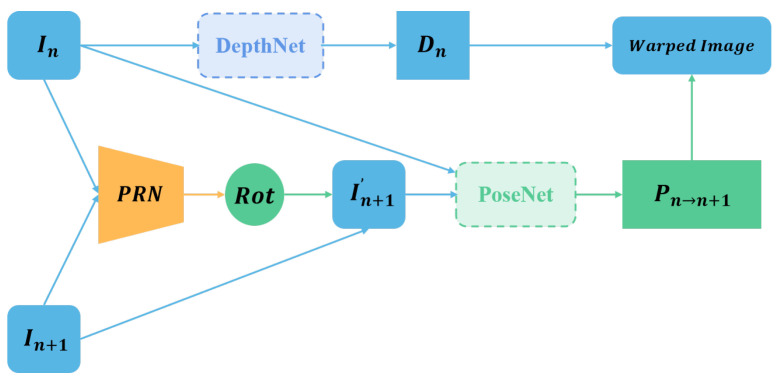
Pipeline of the proposed Pose Rectified Network (PRN). The relative rotational motion between two adjacent frames is estimated by feeding them into the PRN, and then the second frame is rotated to align with the first frame using the estimated rotation, thus removing the rotational motion between the two frames. The aligned frames are then fed into the basic depth estimation pipeline for further learning.

Figure 4 shows the basic framework of the PRN. The pose rectified network operates as follows. First, the PRN network estimates the rotational motion between two frames ($I_{n}$ and $I_{n+1}$), and obtains the rotation matrix Rot. Second, it applies the rotation matrix Rot to warp the second frame ($I_{n+1}$) to align with the first frame ($I_{n}$), and produces a new frame ($I_{n+1}^{\prime }$). This way, the rotational motion between the frames ($I_{n}$ and $I_{n+1}^{\prime }$) is removed and only translational motion remains. Next, it follows the conventional depth estimation steps. The current frame ($I_{n}$) is fed into DepthNet for depth estimation, and the aligned frames ($I_{n}$ and $I_{n+1}^{{}^{\prime }}$) are fed into PoseNet for pose estimation for further learning and training.

**Figure 5:**
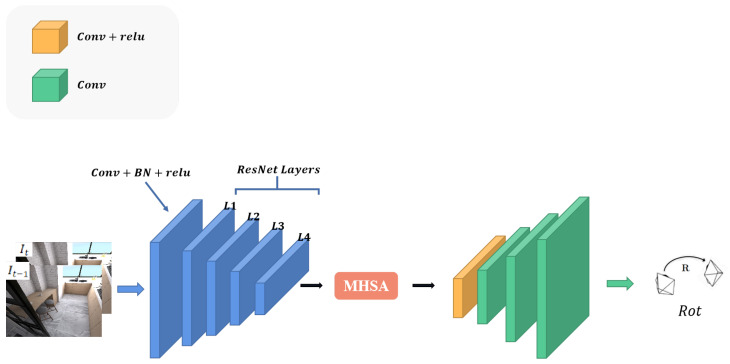
Structure of the proposed Pose Rectified Network (PRN). The input is two adjacent frames, and the output is the relative rotational motion between them. The network is an encoder–decoder architecture with a multi-head self-attention module (MHSA) in the middle.

**Figure 6:**
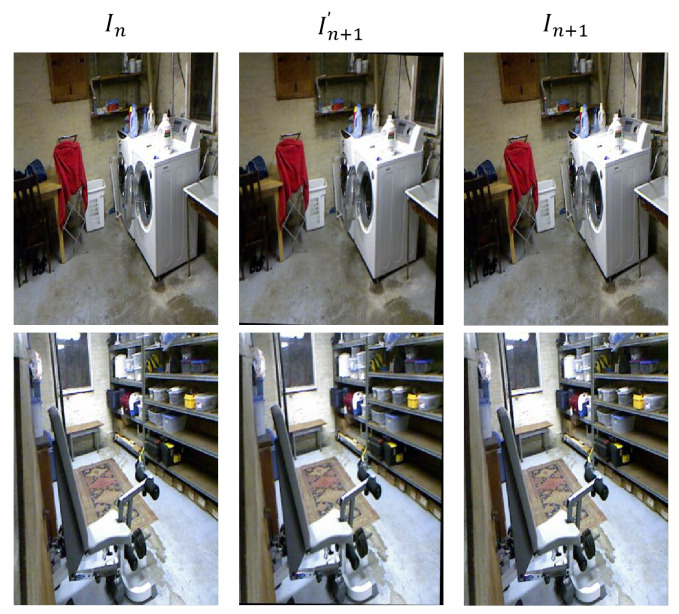
Visualization of PRN warped results. $I_{n}$ and $I_{n+1}$ are two adjacent input frames, and $I_{n+1}^{{}^{\prime }}$ is the reconstruction of $I_{n+1}$ after removing the rotation between $I_{n}$ and $I_{n+1}$ by the PRN network. The black areas in $I_{n+1}^{{}^{\prime }}$ represent the zero-padding process in image warping.

The pose rectified network (PRN) has a similar structure to the pose estimation network, a simple encoder–decoder network, employed in SC_Depthv2 [22], but we improve the structure design of it. To improve the model performance and address the challenges of long-distance dependency and global correlation modeling in image processing, we integrate multi-head self-attention modules (MHSA) into the encoder–decoder architecture. Figure 5 illustrates the structure of the pose rectified network. The output is the camera rotation rather than the six degrees of freedom pose. Moreover, to clearly show the effect of rotation removal, we visualize the images of consecutive frames after removing the rotation. Figure 6 shows the visualization of the PRN warped results.

### 2.4. Multiple Loss Functions

We adopt multiple loss functions [19,20,22,24] as the final loss function to address the issues of non-textured regions and camera pose. The loss function consists of image patch-based photometric consistency loss, Manhattan normal loss, co-planar loss, PRN loss, and edge-aware smoothness loss. The following sections will provide detailed descriptions of each component.

#### 2.4.1. Patch-Based Multi-View Photometric Consistency Loss

Our loss function is based on the photometric consistency loss, a general loss function of self-supervised learning, which uses reprojection to calculate the reprojection error. However, unlike the common loss function in self-supervised learning, we adopt a new image patch-based photometric consistency loss function proposed in P^2^Net [19]. This method uses a support domain-based reprojection to compute the photometric loss, which can handle non-textured region problems more robustly in indoor scenes. The following steps show how to calculate the photometric consistency loss based on image patches.

(2) $$\Omega_{p_{i}}^{t\to s}=KT^{t\to s}D\left(p_{i}\right)K^{-1}\Omega_{p_{i}}^{t},$$

(3) $$\Omega_{p}=\left\{\left(x+x_{p},y+y_{p}\right),x+p\in \left\{-N,0,N\right\}\right.\left.,y_{p}\in \left\{-N,0,N\right\}\right\},$$

where *N* is set to 3. Then, based on this, the improved photometric consistency loss function is

(4) $$L_{SSIM}=SSIM\left(I_{t}\left[\Omega_{p_{i}}^{t}\right],I_{s}\left[\Omega_{p_{i}}^{t\to s}\right]\right),$$

(5) $$L_{L_{1}}={∥I_{t}\left[\Omega_{p_{i}}^{t}\right]-I_{s}\left[\Omega_{p_{i}}^{t\to s}\right]∥}_{1},$$

(6) $$L_{ph}=\alpha L_{SSIM}+\left(1-\alpha \right)L_{L_{1}},$$

where α is set to 0.85.

#### 2.4.2. Manhattan Normal Loss and Co-Planar Loss

Indoor scenes often contain large non-textured regions, which pose a significant challenge for depth estimation. These regions can lead to photometric consistency loss problems and ineffective mismatching. To address this issue, we incorporate the Manhattan normal loss and Co-planar loss proposed in Structdepth [20]. The Manhattan normal loss is

(7) $$L_{norm}=\frac{1}{N_{norm}}\sum M_{p}^{M}M_{p}^{P}\left(1-s\left(n_{p},n_{p}^{align}\right)\right),$$

where $M_{p}^{M}$ represents the Manhattan region, $M_{p}^{P}$ represents the co-planar area, and $N_{norm}$ represents the number of detected pixels located in the Manhattan region. The Co-planar loss is

(8) $$L_{plane}=\frac{1}{N_{plane}}\sum_{p}M_{p}^{P}\left|D_{p}-D_{p}^{plane}\right|,$$

where $N_{plane}$ is the number of pixels in the planar regions $M_{p}$, and $D_{p}^{plane}$ represents the obtained co-planar depth. Here, we adopt the same method as Structdepth [20] for planar region detection. We measure the dissimilarity of planar regions using color and geometry features. Color is compared by the RGB values of the pixels. Geometry is computed by the sum of the differences in normal vectors and distances to the origin of the planes. We apply a graph-based segmentation algorithm [25] to segment the image into planar regions based on the dissimilarity metric. Moreover, this algorithm has a high segmentation efficiency, as it can perform image segmentation in near-linear time, with low added complexity, but still achieve a good improvement of results.

#### 2.4.3. PRN Loss

**Figure 7:**
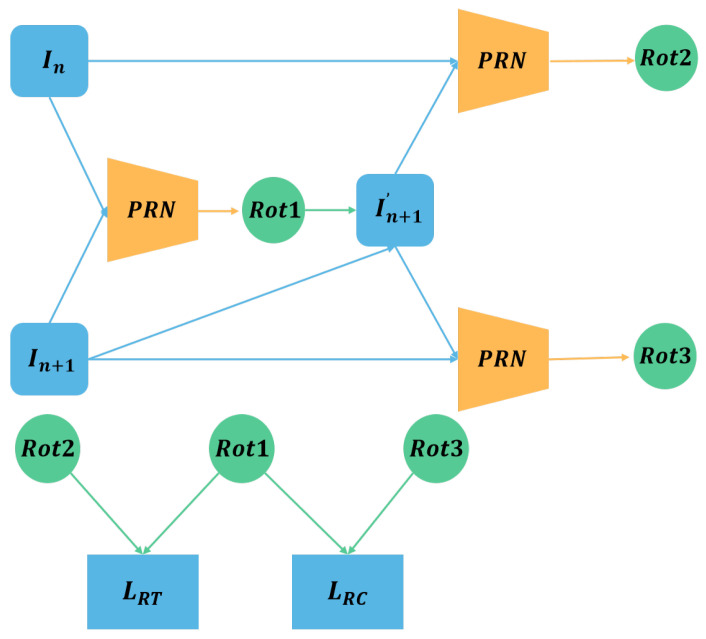
The structure of the Pose Rectified Network (PRN) loss functions. The proposed PRN is used to estimate the rotational motion between two adjacent frames, and the corresponding loss functions are constructed using the Rot1, Rot2, and Rot3 obtained from the PRN to remove the rotational motion between the adjacent frames.

According to the theory and method in SC_Depthv2 [22], which was introduced in Section 2.3, we propose the PRN loss as shown in Figure 7. We use the PRN to generate the image $I_{n+1}^{\prime }$ that removes the rotational motion from the adjacent frame images $I_{n}$ and $I_{n+1}$. In theory, there is no rotational motion between $I_{n}$ and $I_{n+1}^{\prime }$. That is, the Rot2 should be 0 after applying another PRN to $I_{n}$ and $I_{n+1}^{\prime }$. Moreover, the Rot3 obtained by $I_{n+1}$ and $I_{n+1}^{\prime }$ should be equal to the Rot1 obtained in the first step. The structure of the PRN loss is shown in Figure 7. Therefore, we establish the PRN loss as follows:

(9) $$L_{RT}=max\left({∥Rot2∥}_{1}-{∥Rot1∥}_{1}+\delta ,0\right),$$

(10) $$L_{RC}={∥Rot3-Rot1∥}_{1},$$

where δ is set to 0.05.

#### 2.4.4. Edge-Aware Smoothness Loss

Similar to the general unsupervised depth estimation methods, we use the edge-aware smoothness loss function proposed in [24] to ensure smooth depth value changes within the objects:

(11) $$L_{sm}=\left|\partial_{x}d_{t}^{*}\right|e^{-\left|\partial_{x}I_{t}\right|}+\left|\partial_{y}d_{t}^{*}\right|e^{-\left|\partial_{y}I_{t}\right|},$$

where $d_{t}^{*}=d_{t}/\bar{d_{t}}$ is the mean-normalized inverse depth.

#### 2.4.5. Total Loss

Therefore, we can obtain the final loss function form by combining the following loss functions: image patch-based photometric consistency loss, Manhattan normal loss, Co-planar loss, PRN loss, and edge-aware smoothness loss. Different loss functions are used to deal with different problems, as described in the previous sections. Image patch-based photometric consistency loss, Manhattan normal loss, and Co-planar loss are used to handle the non-textured regions problem, and PRN loss is used to handle the camera pose problem. The final loss function can be written as follows:

(12) $$L=L_{ph}+\lambda_{1}L_{sm}+\lambda_{2}L_{RT}+\lambda_{3}L_{RC}+\lambda_{4}L_{plane}+\lambda_{5}L_{norm},$$

where $\lambda_{1}=0.001,\lambda_{2}=0.5,\lambda_{3}=0.1,\lambda_{4}=0.2,\lambda_{5}=0.1.$ Regarding the acquisition of these parameters, we first combine the data from the original papers’ Structdepth [20] and SC_Depthv2 [22], and then scale and recombine them according to the same method as in the original papers. We increase the weights of Manhattan normal loss and Co-planar loss used in Structdepth by a factor of two. Because our improved model has a higher accuracy, adding these two loss functions on this basis will lead to more improvement. The performance of these two loss functions depends on the accuracy of the model. A more accurate model can benefit from using larger weights to impose stronger constraints.

## 3. Experimental Results

### 3.1. Implemention Details

We use P^2^Net [19] without planar consistency loss as our baseline, which is publicly available and built on Pytorch. The depth estimation network employs an enhanced model architecture that integrates MHSA for the depth network. The pose estimation network follows the same methodology as Monodepth2 [17], which infers the relative pose between two image frames given as the input. Our model uses the Adam [26] optimizer and is trained for a total of 50. The learning rate adopts a multi-step learning rate reduction strategy, as in the previous work of Structdepth [20], i.e., the initial learning rate is set to $10^{-4}$, and decays by 0.1 times at the 26th and 36th epochs. In order to speed up training and obtain better results, we train on the pre-trained model [19]. We employ a unique training approach. Initially, we train the network model without Manhattan normal loss and Co-planar loss, with a batch size of 12 for 50 epochs. Subsequently, we add Manhattan normal loss and Co-planarloss and train for an additional 50 epochs with a batch size of 32 to obtain the final results. This is because of previous work [20], which shows that the effectiveness of these two losses depends on the accuracy of depth estimation, as well as to avoid the low quality situation of the initial depth estimation. The training takes about 40 h using NVIDIA GeForce RTX 3090 GPU.

### 3.2. Dataset and Metrics

#### 3.2.1. NYUv2 [23]

We use the NYUv2 [23] dataset, a common benchmark for indoor depth estimation, consisting of 582 video scenes captured indoors with a Microsoft Kinect camera. The original resolution of the images was 640 × 480. We follow the same training segmentation as previous work [18] and use 283 scenes (approximately 230 K images) for training. Based on the method of Structdepth [20], we apply Manhattan normal loss and Co-planar loss to the training set after excluding 18 images that did not have vanishing points. We evaluate our model on the official standard test set of 654 images. We also perform data augmentation on the dataset by randomly flipping, as well as color augmentation. Moreover, we distort all images, crop 16 pixels from each edge, and resize them to 288 × 384 for training. We use the camera intrinsic parameters provided by the official [23] and adjust them according to the cropping and scaling. For training, we use monocular image sequences of five frames each.

#### 3.2.2. Evaluation Metrics

We use two types of evaluation metrics for depth estimation: error and accuracy metrics. The error metrics consist of the root mean squared error (RMSE), mean log10 error (Log10), and absolute relative error (AbsRel). The accuracy metric is the accuracy under the threshold ($\delta^{i}<1.25^{i},i=1,2,3$). Following Monodepth2 [17], we apply a median scaling strategy to account for the scale ambiguity of the self-supervised monocular depth estimation and cap the predicted depth to 10 m.

### 3.3. Results on the NYUv2 [23] Dataset

#### 3.3.1. Quantitative Results

Table 1 shows the quantitative results of our model **PMIndoor** along with the results of supervised and self-supervised methods on the NYUv2 [23] dataset. Our model outperforms several previous self-supervised state-of-the-art methods, namely MovingIndoor [18], TrainFlow [27], P^2^Net [19], and Structdepth [20]. In particular, compared to Structdepth [20], our model achieves lower RMSE (52.8% vs. 54.0%), AbsRel (13.8% vs. 14.2%), and Log10 (5.9% vs. 6.0%) errors and higher $\delta_{1}$ accuracy (82.0% vs. 80.9%), $\delta_{2}$ accuracy (95.6% vs. 95.4%), and $\delta_{3}$ accuracy (98.9% vs. 98.8%) than Structdepth [20]. The reason is that Structdepth [20] only uses Manhattan normal loss and Co-planar loss, while our model employs the proposed PRN network and PRN loss to eliminate the rotational motion between adjacent frames, as well as incorporates multi-head self-attention modules to enhance the model’s accuracy, thereby obtaining better results. Our model also surpasses many previous supervised learning methods [8,28,29,30,31,32,33]. However, there is still a gap between our model and the current state-of-the-art supervised methods. The results of the ablation results are presented in Section 3.4.

#### 3.3.2. Qualitative Results

**Figure 8:**
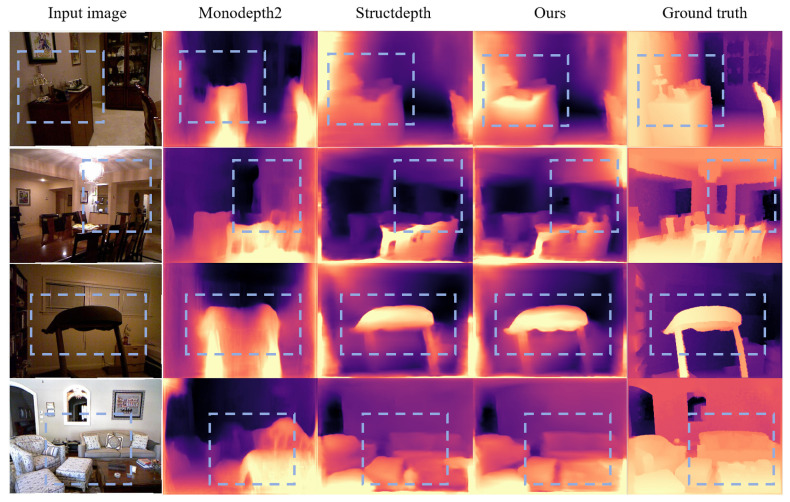
Qualitative comparison on NYUv2 [23]. Images from the left to right are: input, depth from [17,20], **PMIndoor (Ours)**, and Ground truth. Our method achieves a higher accuracy and shows more details.

To demonstrate the effectiveness of our proposed method, we make the visualization shown in Figure 8. We compare different models on the NYUv2 [23] dataset, including the classical network models Monodepth2 [17], Structdepth [20], our model, and we also add the ground truth images as references to better show the validity of our model. Figure 8 shows that our model achieves higher accuracy, especially in the regions marked by the blue dashed boxes. For instance, in the first row, our model can better estimate the contours of the cabinet and the objects on it, while the other two methods perform poorly; for the second row, our model has a clearer estimation of the ceiling and wall, while with the other methods, it is hard to distinguish the estimated results; similarly, for the third row, our method has a very clear contour estimation of the object shown in the image, which is very close to the ground truth; likewise, for the fourth row, our model can better capture the details of the furniture, such as the sofa, table, etc., as indicated by the blue dashed boxes. Thus, it can be seen that our method has a significant improvement over the previous methods and achieves a good effect.

### 3.4. Ablation Studies

We conduct comprehensive experiments and ablation studies on the large indoor benchmark dataset NYUv2 [23] to demonstrate the advantages of our method and the effectiveness of each module. We first perform ablation studies on various network structures to investigate how they affect the experimental results and the overall model performance; we then perform ablation studies on different loss functions to examine how they influence the final results and the overall model performance.

#### 3.4.1. Effects of Network Design for the **PMIndoor** Network

We conduct ablation studies to evaluate the effectiveness of the pose rectified network (PRN) and the multi-head self-attention (MHSA) module. First, we perform experiments without using the PRN and MHSA module as a baseline. For all the experiments, we use all the proposed loss functions except for the PRN loss. The results are presented in Table 2. The first row of Table 2 represents the most basic case, where neither the PRN nor MHSA are applied. The second and third rows represent the cases where the PRN and MHSA are, respectively, added. The last row represents the case where both the PRN and MHSA are integrated. Table 2 indicates that both the PRN and the MHSA module enhance the model performance. The addition of the pose rectified network (PRN) improves the performance of the model on several metrics. The AbsRel is decreased from 0.142 to 0.141, and the RMSE is reduced from 0.540 to 0.538. The $\delta_{1}$ is increased from 81.3% to 81.4%, and the $\delta_{2}$ is increased from 95.4% to 95.5%. The MHSA module also enhances the model’s performance. The AbsRel decreases from 0.142 to 0.140, the Log10 decreases from 0.060 to 0.059, and the RMSE decreases from 0.540 to 0.533. The $\delta_{1}$ increases to 81.8% and the $\delta_{2}$ also increases to 95.5%. When combined with the PRN, these two methods achieve even better results. The $\delta_{1}$ increases to 82.1%, and the $\delta_{2}$ increases to 95.6%. The AbsRel decreases to 0.138, and the RMSE decreases to 53.0%. These are substantial improvements over the baseline.

#### 3.4.2. Effects of the Proposed Losses

To assess the effectiveness of the proposed PRN loss and the impacts of Manhattan normal loss and Co-planar loss, we perform ablation experiments using the same network architecture, namely adding the PRN and MHSA module to the original network framework. The results are shown in Table 3. The first row indicates the case without employing the PRN loss, Manhattan normal loss, and Co-planar loss. The second and third rows indicate the cases where the PRN loss, Manhattan normal loss, and Co-planar loss are separately employed. The last row indicates the case where all the losses are employed, comprising the PRN loss, Manhattan normal loss, and Co-planar loss. The experimental results in Table 3 show that the PRN loss, Manhattan normal loss, and Co-planar loss all improve the model performance. By adding the PRN loss, we lower the AbsRel from 0.147 to 0.146, and the RMSE from 0.560 to 0.556. We also raise the $\delta_{1}$ and $\delta_{2}$ to 80.7% and 95.4%, respectively. The Manhattan normal loss and the Co-planar loss further boost the performance. They reduce the AbsRel to 0.138, and the RMSE to 0.530. They also enhance the $\delta_{1}$ and $\delta_{2}$ to 82.1% and 95.6%, respectively. The combination of these two losses achieves the best results, especially on the RMSE metric, which decreases to 0.528.

### 3.5. Real-Time Performance Comparison

Depth estimation is the process of recovering the depth information of a three-dimensional scene from a single or multiple two-dimensional images. It is an essential component for many applications such as autonomous driving, augmented reality, three-dimensional reconstruction, etc. These applications often demand real-time performance, which requires depth estimation models to be able to produce accurate depth maps with high efficiency. In order to assess the real-time performance of our proposed model, we perform a frame rate (FPS) test and compare it with several other state-of-the-art depth estimation methods. The test results are presented in Table 4.

As shown in the table, our model attains a remarkable frame rate of 55.2 FPS, which makes it feasible for real-world applications. In contrast, the Monodepth2 [17] method lags behind our model in both speed and accuracy aspects. Furthermore, our model preserves a high depth estimation accuracy that outperforms Structdepth [20], while achieving a similar frame rate with it. This indicates that our model has a favorable trade-off between accuracy and efficiency.

## 4. Conclusions

In this work, we propose a novel indoor depth estimation framework **PMIndoor**, which mainly consists of three modules: (a) Pose Rectified Network (PRN): we introduce a Pose Rectified Network (PRN) before the pose estimation network to remove the rotational motion between adjacent frames, which can obtain more accurate pose estimation results and solve the camera pose problem. (b) Multiple Loss Functions: we employ multiple loss functions (including Patch-based Multi-view Photometric Consistency Loss, Manhattan normal loss, Co-planar loss, PRN loss, etc.) to simultaneously address the camera pose problem and non-textured regions. (c) Multi-Head Self-Attention Module: the Multi-Head Self-Attention Module (MHSA) can enable the model to focus on multiple key regions at the same time, enhancing the ability of capturing features at different positions and scales in the image, and improving the expressive and generalization ability of the model. We incorporate the Multi-Head Self-Attention Module (MHSA) into the depth estimation network to improve the accuracy of the model. Experimental evaluations demonstrate the superior performance of our method.

## Figures and Tables

**Table 1:** Comparison of our method to existing supervised and self-supervised methods on NYUv2 [23]. Our method is the best among the self-supervised methods here. ↓ indicates that lower is better; ↑ indicates that higher is better. The best results among supervised and self-supervised methods are in **bold**.

Methods	Supervision	Error ↓			Accuracy ↑		
		**AbsRel**	**Log10**	**RMSE**	$\delta_{1}$	$\delta_{2}$	$\delta_{3}$
Make3D [28]	✓	0.349	-	1.214	44.7	74.5	89.7
Liu et al. [29]	✓	0.335	0.127	1.060	-	-	-
Wang et al. [30]	✓	0.220	0.094	0.745	60.5	89.0	97.0
Eigen et al. [31]	✓	0.158	-	0.641	76.9	95.0	98.8
Chakrabarti et al. [32]	✓	0.149	-	0.620	80.6	95.8	98.7
Li et al. [8]	✓	0.143	0.063	0.635	78.8	95.8	99.1
Laina et al. [33]	✓	0.127	0.055	0.573	81.1	95.3	98.8
VNL [34]	✓	**0.108**	**0.048**	**0.416**	**87.5**	**97.6**	**99.4**
MovingIndoor [18]	✗	0.208	0.086	0.712	67.4	90.0	96.8
TrainFlow [27]	✗	0.189	0.079	0.686	70.1	91.2	97.8
Monodepth2 [17]	✗	0.161	0.068	0.600	77.1	94.8	98.7
P^2^Net(3-frame) [19]	✗	0.159	0.068	0.599	77.2	94.2	98.4
P^2^Net(5-frame) [19]	✗	0.150	0.064	0.561	79.6	94.8	98.6
Structdepth [20]	✗	0.142	0.060	0.540	81.3	95.4	98.8
Baseline (P^2^Net [19] w/o planar loss)	✗	0.166	-	0.612	75.8	94.5	98.5
**PMIndoor (Ours)**	✗	**0.138**	**0.059**	**0.528**	**82.0**	**95.6**	**98.9**

**Table 2:** Ablation results on the network of our **PMIndoor**. ↓ indicates that lower is better; ↑ indicates that higher is better. The best results are in **bold**.

Methods (w/o PRN Loss)	Error ↓			Accuracy ↑		
	**AbsRel**	**Log10**	**RMSE**	$\delta_{1}$	$\delta_{2}$	$\delta_{3}$
Original	0.142	0.060	0.540	81.3	95.4	98.8
PRN-Only	0.141	0.060	0.538	81.4	95.5	98.8
MHSA-Only	0.140	**0.059**	0.533	81.8	95.5	98.9
**Ours (full)**	**0.138**	**0.059**	**0.530**	**82.1**	**95.6**	**98.9**

**Table 3:** Ablation results on losses of our **PMIndoor**. ↓ indicates that lower is better, ↑ indicates that higher is better. The best results are in **bold**.

Methods	Error ↓			Accuracy ↑		
	**AbsRel**	**Log10**	**RMSE**	$\delta_{1}$	$\delta_{2}$	$\delta_{3}$
Original	0.147	0.062	0.560	80.6	95.3	98.8
PRN loss-Only	0.146	0.062	0.556	80.7	95.4	98.8
Manhattan loss + Co-planar loss-Only	**0.138**	**0.059**	0.530	**82.1**	**95.6**	**98.9**
**Ours (full loss)**	**0.138**	**0.059**	**0.528**	82.0	**95.6**	**98.9**

**Table 4:** Real-time Performance Comparison on NYUv2 [23]. ↓ indicates that lower is better; ↑ indicates that higher is better. The best results are in **bold** and the second best are underlined.

Methods	FPS	Error ↓			Accuracy ↑		
		**AbsRel**	**Log10**	**RMSE**	$\delta_{1}$	$\delta_{2}$	$\delta_{3}$
Monodepth2 [17]	45.2	0.161	0.068	0.600	77.1	94.8	98.7
Structdepth [20]	**55.8**	0.142	0.060	0.540	81.3	95.4	98.8
**PMIndoor (Ours)**	55.2	**0.138**	**0.059**	**0.528**	**82.0**	**95.6**	**98.9**

## Data Availability

The data presented in this study are openly available in reference number [23].
